# Downregulation of the silent potassium channel Kv8.1 increases motor neuron vulnerability in amyotrophic lateral sclerosis

**DOI:** 10.1093/braincomms/fcae202

**Published:** 2024-06-11

**Authors:** Xuan Huang, Seungkyu Lee, Kuchuan Chen, Riki Kawaguchi, Ole Wiskow, Sulagna Ghosh, Devlin Frost, Laura Perrault, Roshan Pandey, Joseph R Klim, Bruno Boivin, Crystal Hermawan, Kenneth J Livak, Daniel H Geschwind, Brian J Wainger, Kevin C Eggan, Bruce P Bean, Clifford J Woolf

**Affiliations:** F.M. Kirby Neurobiology Research Center, Boston Children’s Hospital, Boston, MA 02115, USA; Department of Neurobiology, Harvard Medical School, Boston, MA 02115, USA; F.M. Kirby Neurobiology Research Center, Boston Children’s Hospital, Boston, MA 02115, USA; Department of Neurobiology, Harvard Medical School, Boston, MA 02115, USA; F.M. Kirby Neurobiology Research Center, Boston Children’s Hospital, Boston, MA 02115, USA; Department of Neurobiology, Harvard Medical School, Boston, MA 02115, USA; Department of Psychiatry and Semel Institute for Neuroscience and Human Behavior, David Geffen School of Medicine, University of California Los Angeles, Los Angeles, CA 90095, USA; Department of Stem Cell and Regenerative Biology and Department of Molecular and Cellular Biology, Harvard Stem Cell Institute, Cambridge, MA 02138, USA; Department of Stem Cell and Regenerative Biology and Department of Molecular and Cellular Biology, Harvard Stem Cell Institute, Cambridge, MA 02138, USA; F.M. Kirby Neurobiology Research Center, Boston Children’s Hospital, Boston, MA 02115, USA; F.M. Kirby Neurobiology Research Center, Boston Children’s Hospital, Boston, MA 02115, USA; F.M. Kirby Neurobiology Research Center, Boston Children’s Hospital, Boston, MA 02115, USA; Department of Neurobiology, Harvard Medical School, Boston, MA 02115, USA; Department of Stem Cell and Regenerative Biology and Department of Molecular and Cellular Biology, Harvard Stem Cell Institute, Cambridge, MA 02138, USA; F.M. Kirby Neurobiology Research Center, Boston Children’s Hospital, Boston, MA 02115, USA; F.M. Kirby Neurobiology Research Center, Boston Children’s Hospital, Boston, MA 02115, USA; Translational Immunogenomics Lab, Dana-Farber Cancer Institute, Boston, MA 02215, USA; Department of Psychiatry and Semel Institute for Neuroscience and Human Behavior, David Geffen School of Medicine, University of California Los Angeles, Los Angeles, CA 90095, USA; Department of Neurology, Mass General Brigham and Harvard Medical School, Boston, MA 02114, USA; Department of Stem Cell and Regenerative Biology and Department of Molecular and Cellular Biology, Harvard Stem Cell Institute, Cambridge, MA 02138, USA; Department of Neurobiology, Harvard Medical School, Boston, MA 02115, USA; F.M. Kirby Neurobiology Research Center, Boston Children’s Hospital, Boston, MA 02115, USA; Department of Neurobiology, Harvard Medical School, Boston, MA 02115, USA

**Keywords:** motor neuron, neurodegeneration, ion channels, ALS

## Abstract

While voltage-gated potassium channels have critical roles in controlling neuronal excitability, they also have non-ion–conducting functions. Kv8.1, encoded by the KCNV1 gene, is a ‘silent’ ion channel subunit whose biological role is complex since Kv8.1 subunits do not form functional homotetramers but assemble with Kv2 to modify its ion channel properties. We profiled changes in ion channel expression in amyotrophic lateral sclerosis patient–derived motor neurons carrying a superoxide dismutase 1(A4V) mutation to identify what drives their hyperexcitability. A major change identified was a substantial reduction of KCNV1/Kv8.1 expression, which was also observed in patient-derived neurons with C9orf72 expansion. We then studied the effect of reducing KCNV1/Kv8.1 expression in healthy motor neurons and found it did not change neuronal firing but increased vulnerability to cell death. A transcriptomic analysis revealed dysregulated metabolism and lipid/protein transport pathways in KCNV1/Kv8.1-deficient motor neurons. The increased neuronal vulnerability produced by the loss of KCNV1/Kv8.1 was rescued by knocking down Kv2.2, suggesting a potential Kv2.2-dependent downstream mechanism in cell death. Our study reveals, therefore, unsuspected and distinct roles of Kv8.1 and Kv2.2 in amyotrophic lateral sclerosis–related neurodegeneration.

## Teaser

Through RNA sequencing and ion channel profiling, Huang *et al*. discovered that in amyotrophic lateral sclerosis patient–derived motor neurons, reduced Kv8.1 levels heighten cell death susceptibility without altering neuronal excitability. The vulnerability to cell death, at least in part, is contributed to Kv8.1/Kv2.2 interactions, which opens a novel therapeutic opportunity.

## Graphical Abstract

**Graphical Abstract:**
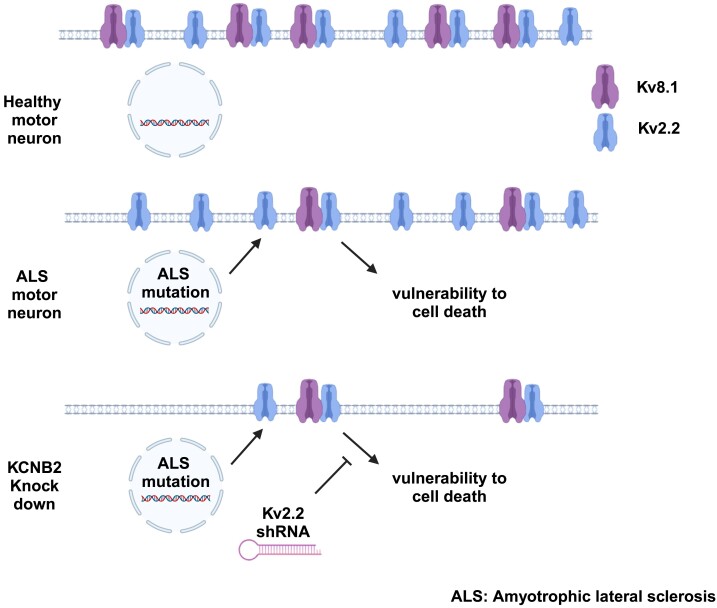

## Introduction

Amyotrophic lateral sclerosis is a devastating neurodegenerative disease that results in a rapid and progressive loss of motor neurons (MNs) in the motor cortex and spinal cord.^1,2^ Advances in induced pluripotent stem cell (iPSC) technology not only enable *in vitro* modelling of amyotrophic lateral sclerosis but also facilitate the discovery of novel molecular targets in diseased neurons.^3^ MNs differentiated from iPSCs derived from patients with sporadic and familial forms of amyotrophic lateral sclerosis recapitulate key hallmarks of the disease, including increased cell death, shorter neurite outgrowth, protein aggregation, endoplasmic reticulum and unfolded protein stress responses and abnormal excitability.^4-7^ Previously, our lab found that a positive feed-forward cycle of endoplasmic reticulum stress and abnormal excitability drives neuronal death in patient-derived MNs harbouring the familial amyotrophic lateral sclerosis A4V mutation in superoxide dismutase 1 (*SOD1^A4V/+^*) and other familial amyotrophic lateral sclerosis MNs.^4,5,8^

Because of the association between abnormal excitability and accelerated disease progression in amyotrophic lateral sclerosis,^9^ we set out to find its molecular basis in MNs with the SOD1(A4V) mutation. However, while we identified a marked downregulation of the silent ion channel subunit KCNV1/Kv8.1 in patient MNs, we found that a loss of KCNV1 did not affect excitability but did increase vulnerability to cell death. We also found that KCNV1 interacts with Kv2.2 that may contribute to amyotrophic lateral sclerosis pathogenesis.

## Materials and methods

### iPSC cultures

The following clonal iPSC lines were used in this study: patient iPSCs [39b SOD1^A4V/+^ (female), Rb9d SOD1^A4V/+^ (female), C9orf72 expansion (male)], their isogenic control iPSCs (39b-cor SOD1^+/+^, Rb9d-cor SOD1^+/+^, C9orf72 corrected) and a healthy wild-type line 11a. iPSCs were maintained with mTeSR1 media (StemCell Technologies) or StemFlex media (Gibco #A3349401) on culture dishes coated with Matrigel (BD Biosciences). Media was changed daily, and cells were typically passaged by accutase. All cell cultures were maintained at 37°C, 5% CO_2_.

### HB9::GFP transfection using zinc finger nuclease

For transfection of a HB9::GFP reporter into each of the four iPSC lines, a 1 kb HB9 promoter fragment (gift from Hynek Wichterle) controlling the expression of myristoylated GFP was inserted into a donor plasmid specific for the AAVS1 locus (Sigma). Subsequently, 2.5 million iPSCs were treated with accutase and electroporated using the Neon Transfection System (100 μL tip; 1600 V voltage, 20 ms width, 1 pulse; Life Technologies) with 2 μg of AAVS1 ZFN plasmid and 6 μg of the modified AAVS1 donor plasmid. After nucleofection, cells were plated on Matrigel with mTeSR1 in the presence of 10 μM ROCK inhibitor. After 48-h puromycin selection, surviving clonal colonies were individually passaged and genomic DNA was extracted. Polymerase chain reaction (PCR) was used to confirm proper targeting of the cassette. Expression of the reporter was verified using expression of the GFP and the MN marker Isl1.

### MN differentiation

MN differentiation for patch-seq and patch-quantitative PCR (qPCR) was carried out as described previously^10^ with modifications^11^ in a 24-day protocol based on initial neuralization with SB431542 (10 μM, Sigma Aldrich) and dorsomorphin (1 μM, Stemgent) and MN patterning with retinoic acid (1 μM, Sigma) and a small smoothened agonist 1.3 (1 μM, Calbiochem). Differentiated MNs were dissociated using accutase, filtered with a 70 µm filter and isolated by activated flow cell sorting using HB9::GFP expression. MNs were plated in the presence of P0 mouse glial cells and were maintained in Neurobasal media; supplemented with N2 and B27 (Invitrogen) and 10 ng/mL each of brain-derived neurotrophic factor, glial cell line–derived neurotrophic factor and ciliary neurotrophic factor (R&D) and ascorbic acid (0.4 μg/mL, Sigma); and fed every 2–3 days.

MN differentiation for cell death assays and reverse transcription (RT)-qPCR was carried out with a modified 14-day protocol described in Klim *et al.*^12^ Briefly, iPSC were dissociated using accutase and plated with 1.5 million cells per 10 cm dishes coated with Geltrex. One day after plating, the medium was changed to differentiation medium (half Neurobasal and half DMEM/F12 supplemented with B27 and N2 supplements, GlutaMax and non-essential amino-acids) from StemFlex™ Medium (Gibco). SB431542 of 10 µM, 100 nM LDN-193189, 1 µM retinoic acid and 1 µM smoothened agonist were treated on Days 0–5, and 5 µM DAPT, 4 µM SU-5402, 1 µM retinoic acid and 1 µM smoothened agonist were treated on Days 6–14. The differentiated cells were dissociated using accutase and then filtered with a 70 µm filter. The single-cell suspension was incubated with PE Mouse Anti-Human CD56 (BD Biosciences #555516) and then Anti-R-Phycoerythrin Magnetic Particles (BD Biosciences #557899) in magnetic cell separation buffer (phosphate-buffered saline with 2 mM ethylenediaminetetraacetic acid and 0.5% bovine serum albumin) on ice. The sorted MNs were plated in the presence of P0 mouse glial cells and were maintained in the same medium and condition as described above.

### Immunocytochemistry

Cell cultures were fixed in 4% paraformaldehyde for 15 min at 4°C, permeabilized with 0.2% Triton X in phosphate-buffered saline for 45 min and blocked with 10% donkey serum in phosphate-buffered saline with Triton X-100 (Triton 0.1%). Cells were then incubated in primary antibody overnight and secondary antibodies for 1 h in 2% donkey serum in phosphate-buffered saline with Triton X-100 after several washes in between. The following antibodies were used: primary antibodies, human nuclei (1:1000, Millipore), Islet1 (1:200, DSHB, 40.2D6) and MAP2 (1:10000, Abcam ab5392), and secondary antibodies Alexa Fluor (1:1000, Life Technologies) and DyLight (1:500, Jackson ImmunoResearch Laboratories). Images were acquired and analysed by the ArrayScan™ XTI (Thermo Fisher). To induce cell death, MN–mouse glia co-culture were maintained for 3–4 weeks and then treated with 10 µM MG132 for 48 h before fixation and staining.

### Electrophysiology

Current clamp of iPSC-derived MNs: Purified and differentiated neurons on P0 mouse glial cells were identified microscopically, and whole-cell current clamp recordings were conducted using a MultiClamp 700B amplifier. Data were digitized with a Digidata 1440A A/D interface and recorded using pCLAMP10 (Molecular Devices). Borosilicate glass pipettes were pulled on a P-97 puller (Sutter Instruments). The external solution consisted of 140 mM NaCl, 5 mM KCl, 2 mM CaCl_2_, 2 mM MgCl_2_, 10 mM HEPE (4-(2-hydroxyethyl)-1-piperazineethanesulfonic acid) and 10 mM D-glucose, pH 7.4, adjusted with NaOH. The internal solution consisted of 135 mM K-gluconate, 10 mM KCl, 1 mM MgCl_2_, 5 mM EGTA (ethylene glycol bis(2-aminoethyl)tetraacetic acid) and 10 mM HEPE (4-(2-hydroxyethyl)-1-piperazineethanesulfonic acid). Fast and slow capacitance transients and whole-cell capacitance were compensated using the automatic capacitance compensation in voltage clamp mode on the MultiClamp 700B. After currents were injected to bring the membrane potential to −60 mV in current clamp mode, a depolarizing current ramp (0–700 pA in 1 s) or series of steps (500 ms steps from 0 to 100 pA) were applied to measure the firing frequency and the action potential characteristics. Spikes were counted using a criterion of a peak voltage > −10 mV and amplitude > 20 mV. Frequency of firing was calculated from all the spikes during stimulation and instant frequency from the first two spikes. Action potential characteristics including trough voltage were analysed from the first action potential waveform.

### Singl-cell collection

For patch-qPCR, single neurons were picked immediately after recording, transferred into 8-well strip tubes containing 5 μL lysis buffer (CellsDirect™ One-Step RT-qPCR Kit, #11753100) and then placed on dry ice. Tubes were kept at −80°C until the next pre-amplification step. For patch-seq, the single neurons were collected in 5 μL of 1× TCL buffer (Qiagen, #1031576) in each well of 96-well PCR plates (Eppendorf, #951020401) on dry ice and then kept at −80°C until complementary DNA (cDNA) construction.

### Single-cell RNA sequencing

Single-cell RNA sequencing (RNA-seq) libraries were generated using the Smart-seq2 protocol^13^ with minor modifications.^14^ All libraries were prepared by the Broad Technology Labs and sequenced at the Broad Genomics Platform. Briefly, total RNA from single cells was purified using RNA-SPRI beads. Poly(A)+ mRNA was converted to cDNA and amplified. cDNA was subjected to transposon-based fragmentation using dual-indexing to barcode each transcript fragment with a combination of barcodes specific to a single cell. Barcoded cDNA fragments were then pooled for sequencing. Sequencing was carried out as paired-end 2 × 25 bp with additional eight cycles for each index. To obtain expression values for each cell, the data were demultiplexed and aligned to the human genome (hg19) using Tophat version 2.0.10 with default settings.^15^ Transcripts were quantified by the BTL computational pipeline using Cuffquant version 2.2.1,^16^ and raw counts of reads mapped per gene were extracted from aligned bam using FeatureCounts (subread-1.5.0-p1) with default settings. For visualization purposes, expression levels were converted to log-space by taking the log2(FPKM + 1) or by normalizing read counts to transcripts per million using log2(TPM + 1). To identify genes that are differentially expressed between distinct electrophysiological classes or genotypes, we first filtered cells with poor electrophysiological recordings or low sequencing quality (library size greater or less than three median absolute deviations from the median). Single-cell differential expression analysis was performed using SCDE (single-cell differential expression)^17^ with default settings and batch effects corrected for when calculating expression differences.

### Single-cell RT-qPCR: primer design

DELTAgene assays (Fluidigm) were designed for 279 genes (listed in the Supplementary Table 3), including housekeeping genes (GAPDH and ACTB), MN markers (MNX1, CHAT, ISL1 and SLC18A3), a glial cell marker (mGfap), voltage-gated ion channels and ligand-gated ion channels. The assays are designed to cross an intron. A nested primer strategy (outer and inner pairs of forward and reverse primers) was utilized. Outer primer pairs were used for the pre-amplification, and inner primer pairs were used for RT-qPCR in the Biomark. For outer primers, the oligos were synthesized by IDT and dissolved at a concentration of 200 μM in H_2_O, and 267 gene primer pairs were mixed in a 15 mL falcon tube at a final concentration of 200 nM each primer. For inner primers, the oligos were synthesized by IDT and dissolved at a concentration of 50 μM in H_2_O. To make 10 μL of 20 μM primer pairs per each gene in V-shaped 96-well plate (Eppendorf, #951020401), 4 μL of forward and reverse primers were added to 2 μL1 H_2_O.

### Single-cell RT-qPCR: cDNA synthesis and pre-amplification

We used a protocol adapted from Fluidigm (Application Note MRKT00075e) that combines reverse transcription and pre-amplification (called reverse transcription-specific target amplification) using CellsDirect™ One-Step RT-qPCR Kit. A mixture consisting of 2.8 μL of multiplex outer primers plus 0.2 μL of the enzyme mix (SuperScript® III Reverse Transcriptase and Platinum® Taq DNA Polymerase) was added to each single cell in lysis buffer in the 8-well strips. Following centrifugation, the 8-well strips were moved to a thermal cycler and subjected to the following protocol: 50°C, 60 min for reverse transcription; 95°C, 2 min for Taq enzyme activation; and 20 cycles of 95°C, 15 s and 60°C, 4 min. The reactions were kept at −80°C until the next tests.

To determine optimal dilution for following single-cell qPCR, a few randomly picked reactions were diluted in H_2_O at 25-, 50-, 75- and 100-fold and tested with GAPDH, a housekeeping gene, by qPCR. A dilution of 25-fold was selected for subsequent experiments. All the reactions were diluted and tested with GAPDH, a positive control, and mGfap, a negative control, to determine whether we had properly picked neurons without glial cells.

### Single-cell RT-qPCR: data acquisition and analysis

Diluted reactions were analysed by qPCR using 96.96 Dynamic Array™ Integrated Fluidic Circuits (IFCs) and the Biomark™ HD system from Fluidigm. The IFCs were processed, and the instruments were operated in the BCH IDDRC Molecular Genetics Core Facility according to the manufacturer’s procedures for analysing DELTAgene assays. Three IFCs were used to analyse the 96 samples for the total of 267 assays, and all IFCs included housekeeping genes, GAPDH and ACTB, and a blank. In single-cell qPCR analysis, a Ct of 30 was used as the background value for all real-time signals. Expression levels were calculated by 30 Ct value, assigning a value of 0 when Ct > 30. Hierarchical clustering and heat map were generated with R software.

### CRISPR knockout generations for KCNV1/Kv8.1

39b-cor SOD1^+/+^ iPSCs were maintained in Geltrex-coated 10 cm dishes in StemFlex™ Medium (Gibco, #A3349401) at 37 °C and 5% CO.^2^ Two single guide RNA CUGGACUCGCCGCUGGACAG and GGGUUAGAGAUGCCUUCCAG were designed and synthesized from Synthego to create INDEL in KCNV1. 39b-cor SOD1^+/+^ iPSCs were dissociated into single-cell suspension using accutase with 10 µM Y-27632. One million cells were nucleofected with 1.2 µL of 20 µM Cas9-2NLS and 9 µL of 30 µM each single guide RNA using Nucleofector™ device (programme CB150, Lonza) according to the manufacturer’s instructions. Transfected cells were plated on Geltrex-coated 6-well plates in StemFlex™ Medium with 10 µM Y-27632. After 2 days, the cells were dissociated into single cells and plated at a density of 2000 cells/10 cm dish. One week after transfection, single colonies were picked and sequenced to confirm INDEL. Forward and reverse primers were 5′TGCGCCAAGGAGAGGTAA3′ and 5′TCGCTGCAGAAGACACTAGA3′, respectively.

### RNA extraction and RT-qPCR

RNA was extracted from MN culture using RNeasy Micro Kit (Qiagen), and then, cDNA was generated using SuperScript Vilo Synthesis Kit (Invitrogen) following manufacturer’s manuals. qPCR was performed on Applied Biosystems 7500 machine (Life Technologies) using Fast SYBR Green Master Mix (Roche).

### RNA-seq analysis

RNA-seq libraries were prepared using the TruSeq Stranded RNA Kit with Ribo-Zero Gold to enrich messenger RNA (mRNA). The libraries were then sequenced on a NovaSeq6000 platform, generating paired-end reads of 100 base pairs in length. To ensure the accuracy of downstream analysis, the reads were classified into five categories using Xenome (v1.0.0), a tool designed to remove reads derived from unwanted sources such as contaminating DNA or RNA from other species. Reads classified as human were then aligned to the human genome (Hg38) using STAR aligner (v2.7.5c). Average input read counts were 31.00 ± 3.61 M(SD) and average percentage of uniquely aligned reads were 88.04 ± 0.03(SD)%. Total counts of read fragments aligned to known gene regions within the human ensemble gene model annotation (GRCh38) are used as the basis for quantification of gene expression. Fragment counts were derived using HTSeq program (ver 0.12.4). Quality control measures were performed to assess the quality of the data, including base quality, mismatch rate and mapping rate to the whole genome. Additionally, repeats, chromosomes, key transcriptomic regions (exons, introns, UTRs and genes), insert sizes, AT/GC dropout, transcript coverage and GC bias were assessed to identify potential issues in the library preparation or sequencing. To identify genes that were differentially expressed between conditions, lowly expressed genes were removed and genes with counts per million (CPM) > 0.15 in at least three samples were selected for downstream analysis. Differential expression analysis was conducted using the Bioconductor package EdgeR (ver 3.14.0), which uses a negative binomial model to account for the variability in the data. Expressed genes were sorted by directional pVal and used as input for Gene Set Enrichment Analysis. MsigDB (ver7.0) was used as reference gene set. Raw and processed data were deposited within the Gene Expression Omnibus repository (www.ncbi.nlm.nih.gov/geo).

### Statistical analysis

Students’ *t*-test was performed using GraphPad.

## Results

**Figure 1:**
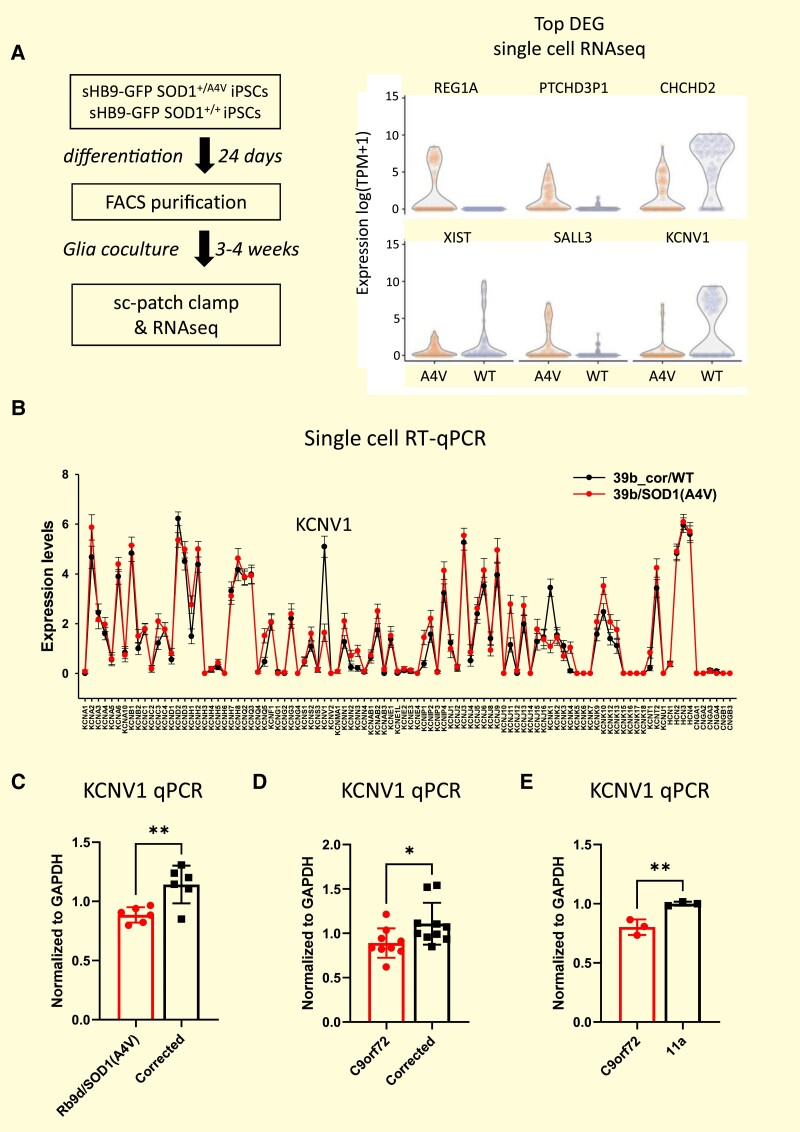
**Single-cell RNA-seq analysis of 39b *SOD1^A4V/+^* and 39b-cor *SOD1^+/+^* MNs reveals KCNV1/Kv8.1 downregulation in mutant MNs. (A)** Single-cell patch-seq scheme and top differential expressed genes identified between 39b *SOD1^A4V/+^* and isogenic control 39b-cor *SOD1^+/+^* MNs. Single-cell RNA-seq collected from total 181 neurons (*N* = 43∼47 for each 39b and 39b-cor in each two different experimental batches) was analysed. **(B)** Comparison of K^+^ channel expression between 39b *SOD1^A4V/+^* and 39b-cor *SOD1^+/+^* MNs, determined by single-cell multiplex RT-qPCR with ion channel probes. qPCR expression data from 128 single neurons (*N* = 64 for each 39b and 39b-cor) were analysed. Average expression levels are connected to visualize differences. **(C)** KCNV1 is also downregulated in MNs from another *SOD1^A4V/+^* patient’s stem cell line (RB9d SOD1^A4V/+^) compared to their isogenic control, as measured by RT-qPCR (*n* = 6). **(D)***KCNV1* mRNA is downregulated in C9orf72 repeat expansion patient stem cell–derived MNs compared to its isogenic control (*n* = 9–10 different 4-week co-culture replicates). **(E)***KCNV1* mRNA is also downregulated in another C9orf72 repeat expansion patient stem cell–derived MNs compared to healthy control line (11a)–derived MNs measured by RT-qPCR (*n* = 3 different 4-week co-culture replicates). Statistical significances by Student’s *t*-test (*P ≤ 0.05, **P ≤ 0.01).

To explore the cellular pathways involved in the hyperexcitability component of amyotrophic lateral sclerosis pathogenesis, we employed a ‘patch-seq’ strategy that enables both neuronal excitability and gene expression to be measured simultaneously at a single cell level.^8^ Compared to prior studies where a heterogeneous population of differentiated neurons were used and neuronal maturity not characterized,^4,9^ we focused on a homogenous population of mature MNs that fire action potentials robustly. We introduced a GFP reporter under control of the Hb9 promoter into *SOD1^A4V/+^* iPSC lines and their isogenic controls^4^ using an AAVS1-sHb9-GFP plasmid. The MNs were FACS purified after differentiation. Immunostaining for ISL1 (a MN marker) and MAP2 (a neuronal marker) revealed that most of the neurons had a MN identity (91.5 ± 1.4% of 39b SOD1^A4V/+^ neurons were ISL1+ MAP2 + and 92.7 ± 1.7% of 39b-cor SOD1+/+; Supplementary Fig. 1A). To accelerate neuronal maturation, we cultured the purified MNs with glia for 3 weeks and then performed whole-cell current clamp recordings followed by single MN isolation for single-cell RNA-seq (Smart-seq2; Fig. 1A). Two independent batches comprising a total of 181 MNs were analysed, and ∼8000 genes per neuron were detected (Supplementary Table 1).

There were no significant differences in housekeeping, MN or neuronal marker gene expression between the SOD1 mutant and control MNs (Supplementary Fig. 1B). A significant difference in the expression of 63 genes was however detected by the single-cell patch-seq analysis of 39b *SOD1^A4V/+^* and 39b-cor *SOD1^+/+^* MNs (Fig. 1A), including a marked downregulation of KCNV1 (Supplementary Table 2). We independently confirmed this finding with a single-cell patch-RT-qPCR analysis focused specifically on the differential expression of 279 ion channel genes (Fig. 1B; Supplementary Table 1). There was no difference in voltage-gated Na^+^ or Ca^2+^ channel expression (Supplementary Fig. 2), and the expression signatures of most K^+^ channel subtypes were also generally very similar between diseased and healthy MNs. However, KCNV1/Kv8.1 was significantly downregulated in 39b *SOD1^A4V/+^* MNs relative to their isogenic controls (39b-cor *SOD1^+/+^* MNs) both in the single-cell patch-RT-qPCR analysis and the patch-seq study (Fig. 1B).

To determine whether KCNV1 downregulation is consistent across different cell lines, an independent patient-derived iPSC line with the same mutation, RB9d *SOD1^A4V/+^*, and its isogenic control RB9d-cor *SOD1^+/+^* were also investigated. RB9d *SOD1^A4V/+^* MNs showed a similar downregulation of KCNV1/Kv8.1 compared to the control RB9d-cor *SOD1^+/+^* neurons (Fig. 1C). To test if this observation also holds for other amyotrophic lateral sclerosis mutations, we examined KCNV1 expression in MNs differentiated from iPSCs harbouring C9orf72 hexanucleotide repeat expansions. We also found KCNV1 downregulation in C9orf72 repeat expansion iPSC MNs compared to their isogenic control MNs (provided by Dr. Coppola; Fig. 1D), as well as in another independent C9orf72 mutant line compared to healthy controls (Fig. 1E). The downregulation of KCNV1 in diverse amyotrophic lateral sclerosis MN models and lines suggests that this could contribute to disease progression.

**Figure 2:**
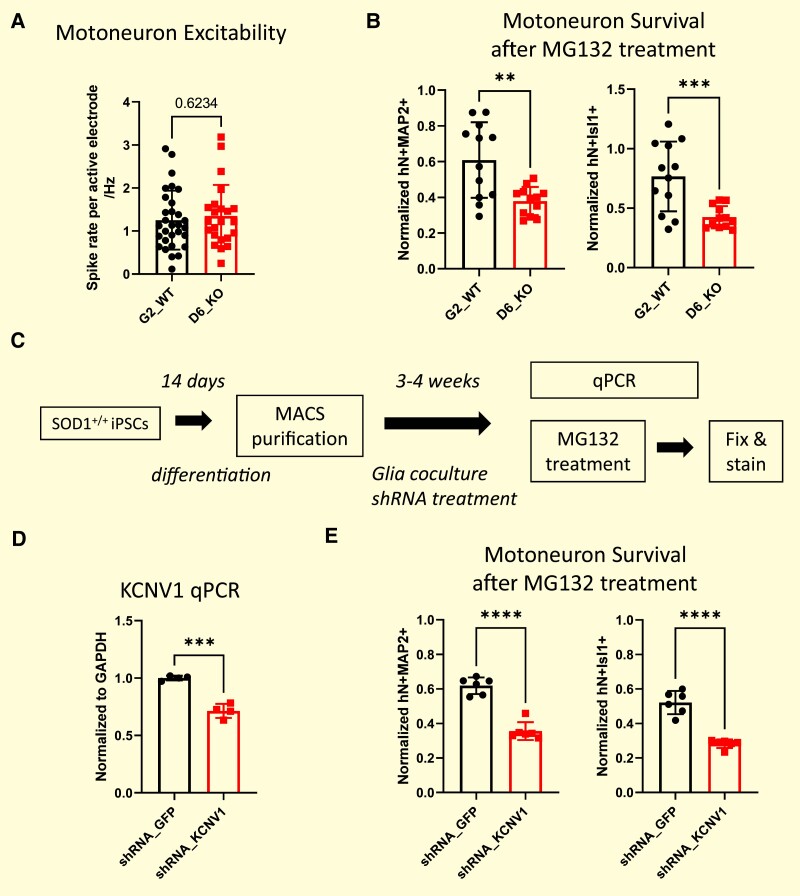
**Loss of *KCNV1*/Kv8.1 increases MG132-induced neuronal cell death. (A**) Spontaneous activity of KCNV1 CRISPR knockout (D6) and control (G2) MNs recorded on a multiple electrode array. Average spike rates of active electrodes are measured (*n* = 23–30 different 4-week co-culture replicates and average spike rates calculated from eight electrodes for each co-culture). **(B)** After culturing MNs with glia for 4 weeks, MG132 (10 µM) was added into the culture to induce cell death. Forty-eight-hour post-treatment, the cell culture was fixed and stained with MN markers: hN (human nucleus marker), MAP2 (mature neuron marker), and Isl1 (MN nucleus marker). The number of hN^+^Isl1^+^ cells or hN^+^MAP2^+^ cells were quantified and normalized to dimethylsulfoxide-treated control for each cell line (*n* = 12 different co-culture replicates from two independent differentiations, with *n* = 6 for each batch; average count calculated from >10 image fields for each co-culture replicate). A greater cell death occurred in KCNV1 knockout than control MNs. **(C)** Outline of 2D differentiation, NCAM magnetic cell separation, glia co-culture, shRNA treatment, followed by RT-qPCR analysis and cell death assays. **(D)** KCNV1 expression reduction in non-mutant 39b-cor *SOD1^+/+^* MNs treated with a lentivirus encoding KCNV1 shRNA (*n* = 4 different co-culture replicates from one batch of differentiation) compared to those treated with a control GFP targeting shRNA. **(E)** 39b-cor *SOD1^+/+^* MNs treated with KCNV1 shRNA show less survival after MG132 treatment than control GFP shRNA–treated MNs (*n* = 6 different co-culture replicates; average count calculated from >10 image fields for each co-culture replicate). Statistical significance by Student’s *t*-test (**P ≤ 0.01, ***P ≤ 0.001, **** *P* ≤ 0.0001).

KCNV1/Kv8.1 is a ‘silent’ potassium channel subunit that, while it has a structure typical of voltage-activated potassium channels, cannot form functional homomeric channels. Instead, when it forms heteromeric channels with Kv2, it downregulates Kv2 channel density and modulates channel gating.^18,19^ To study the role of KCNV1/Kv8.1 in healthy MNs, we produced a knockout of KCNV1 by CRISPR gene editing in the control 39b-cor *SOD1^+/+^* iPSCs (Supplementary Fig. 3A). Introduction of INDEL mutations resulted in early stop codon formation at the N-terminal that significantly reduced KCNV1 expression (Supplementary Fig. 3B). The KCNV1 knockout MNs did not exhibit altered spontaneous action potential firing, as assessed by multi-electrode array recordings, when compared to KCNV1 intact controls, indicating that loss of KCNV1 does not directly affect MN excitability (Fig. 2A).

Amyotrophic lateral sclerosis is characterized by a progressive loss of MNs, and we wondered whether KCNV1 may impact neuronal survival. To test this, we induced cell death using MG132, a proteasome inhibitor that promotes mutant SOD1 protein aggregation.^5^ KCNV1 knockout MNs exhibited a higher rate of MG132-induced cell death when compared to non-CRISPR-edited controls (Fig. 2B). To independently confirm this result, we also knocked down KCNV1 expression in control 39b-cor *SOD1^+/+^* MNs to mimic the KCNV1 downregulation in amyotrophic lateral sclerosis, using two different small hairpin RNAs (shRNAs) targeting KCNV1/Kv8.1, and found that the knockdown of KCNV1 in these healthy control MNs increased MG132-induced cell death significantly, while not affecting basal survival (Fig. 2C–E; Supplementary Fig. 4). A downregulation of KCNV1 in MNs, increases, therefore, their vulnerability to cell death without changing their excitability.

**Figure 3:**
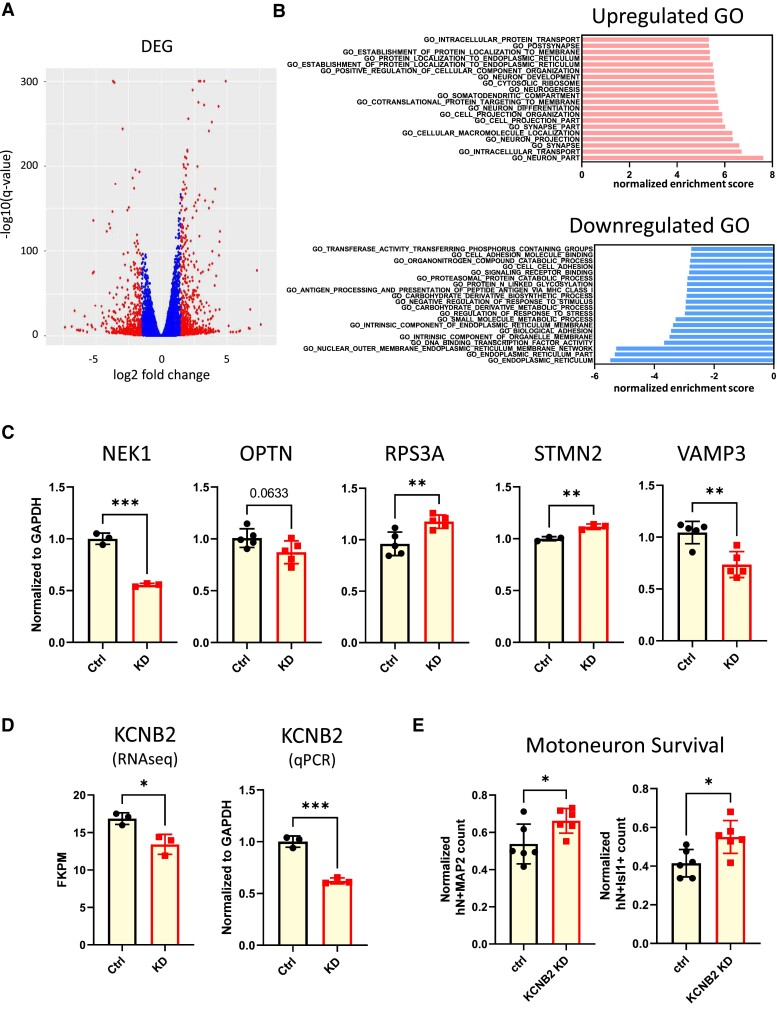
**Loss of KCNV1/Kv8.1 disrupts MN metabolism and lipid/protein transport through the KCNB2/Kv2.2 channel. (A)** Volcano plot of differentially expressed genes in *KCNV1* knockdown MNs. NCAM-sorted MNs were treated with a lentivirus-expressing KCNV1 targeting shRNA or control GFP targeting shRNA and samples collected for bulk-seq analysis after 3 weeks (*n* = 3 different co-culture replicates). The distribution between log2(fold change KD/WT) and -log10(*q*-value) are plotted. Blue, log2(fold change) < 1.5; red, log2(fold change) ≥ 1.5. **(B)** Top enrichment analysis of Gene Ontology in KCNV1 knockdown MNs. *X*-axis: normalized enrichment score. **(C)** RT-qPCR validation of five genes in KCNV1 knockdown MNs (*n* = 3–5 different co-culture replicates). **(D)** Reduced expression of the KCNB2/Kv2.2 transcript detected by RNA-seq (left, *n* = 3 different co-culture replicates) and RT-qPCR (right, *n* = 3 different co-culture replicates) in KCNV1 knockdown MNs. **(E)** 39b *SOD1^A4V/+^* MNs treated with *KCNB2* shRNA show increased survival after MG132 treatment (*n* = 6 different co-culture replicates; average count calculated from >10 images fields for each co-culture replicate). Statistical significance by Student’s *t*-test (*P ≤ 0.05, **P ≤ 0.01, ***P ≤ 0.001, ****P ≤ 0.0001).

How does a reduction of KCNV1/Kv8.1 contribute to amyotrophic lateral sclerosis–related MN cell death? To explore this, we studied transcriptomic alterations caused by a reduction in KCNV1 expression. Many genes were significantly differently expressed in KCNV1 shRNA knockdown MNs compared to their controls (Fig. 3A; Supplementary Table 3), including genes involved in lipid metabolism, protein translation and membrane transport pathways (Supplementary Fig. 5A). Gene sets related to the endoplasmic reticulum membrane, metabolism and catabolism were among the top sets downregulated, while gene sets associated with neuron projection and intracellular transport were among the top sets that were upregulated (Fig. 3B). We validated the differential expression of several of the genes (Fig. 3C), including NEK1 and OPTN, two amyotrophic lateral sclerosis–associated genes that participate in proteostasis regulation^20,21^; RPS3A, a ribosomal and chaperone protein that counteracts α-synuclein aggregation^22^; STMN2, a microtubule-associated protein involved in TDP43 pathology^12,23^; and VAMP3, a vesicle-associated membrane protein that directs transport of proteolipid proteins.^24^ Collectively, the transcriptomic analysis identified several overlapping molecular pathways between amyotrophic lateral sclerosis and KCNV1 knockdown MNs. Expression of KCNV1 was also reduced by tunicamycin treatment that interferes with protein glycosylation and results in misfolding, suggesting a possible involvement of KCNV1 in intracellular protein transport (Supplementary Fig. 5B).

KCNV1/Kv8.1 forms heteromers with Kv2 channels, and we therefore examined if Kv2 was also involved in the MN cell death induced by a reduction of KCNV1 expression. While channels formed by Kv2 subunits regulate neuronal excitability,^25,26^ they also have non-ion channel functions, including chaperoning proteins to the plasma membrane^27^ and coupling the plasma membrane to the endoplasmic reticulum membrane,^28,29^ as well as producing pro-apoptotic effects under conditions of oxidative stress.^30-32^ Recent studies show that endoplasmic reticulum–plasma membrane–located Kv2 ion channels regulate endoplasmic reticulum Ca^2+^ uptake and release in neurons^33-35^ as well as lipid metabolism^36^ that could impact multiple biological processes, including synaptic transmission, receptor signalling, membrane trafficking and cytoskeletal dynamics.^37^

We found that the expression of KCNB2, which encodes the Kv2.2 ion channel, was significantly decreased in KCNV1 knockdown MNs (Fig. 3D). Potentially, this change could be either protective or pathogenic. To explore this, we knocked down KCNB2/Kv2.2 expression in 39b *SOD1^A4V/+^*ALS MNs and then induced cell death using MG132. We found that the survival of SOD(A4V) amyotrophic lateral sclerosis MNs was significantly increased by KNCB2 shRNA knockdown when compared to SOD1(A4V) MNs with no KNCB2 knockdown (Fig. 3E). Suppression of Shab, a *Drosophila* gene orthologous to several human genes including KCNB2 (https://ncbi.nlm.nih.gov/gene/38352), was recently reported to rescue the eye degeneration found in a C9orf72 expansion *Drosophila* model.^38^ We also observed a trend of increased survival in C9orf72 patient MNs when the expression of KCNB2 was suppressed (Supplementary Fig. 6). It is therefore possible that a reduction in KCNV1/Kv8.1 increases MN vulnerability through alterations in Kv2.2 ion channels and that the transcriptional downregulation of Kv2.2 in KCNV1/Kv8.1 knockdown MNs is, therefore, a protective compensatory response.

## Discussion

Potassium ion channels are the most diverse and widely distributed ion channels in mammals and play critical roles in controlling membrane excitability. Recently, other functions of potassium channels have been reported, suggesting they modulate cellular Ca^2+^ metabolism,^33,34^ lipid metabolism,^36^ apoptosis^39^ and neuron–microglia interactions.^40^ Our data now reveal that an experimental reduction in the expression of the silent Kv8.1 subunit increases MN vulnerability and that vulnerable amyotrophic lateral sclerosis MNs have a reduction in Kv8.1 expression. We suggest this may occur through a loss of interaction with those Kv2.2 channels that could lead to the dysregulation of multiple pathways, including lipid metabolism and membrane transport. While the expression of Kv8.1 and Kv2.2 have not been reported to be altered in SOD(A4V) transgenic mice, KCNV1/Kv8.1 is reduced in the motor cortex of sporadic amyotrophic lateral sclerosis patients.^2^

The precise underlying molecular machinery of Kv8.1–Kv2.2 interactions in amyotrophic lateral sclerosis MNs need now to be defined, since this may open opportunities for novel therapeutic interventions. We cannot completely rule out a potential involvement of the ion-conducting functions of Kv2 in the MN disease state. Kv2 channels are important in regulating neuronal firing,^41^ and BACE2, a protease that generates amyloid β, cleaves the C-terminal of Kv2, resulting in reduced K currents and increased neuronal apoptosis.^42^ Kv2 inhibition also protects against the degeneration of pancreatic β cells.^43^ Nevertheless, we did not observe any differential effects of the Kv2 blocker GXTX^44^ on the KCNV1 knockout MNs (Supplementary Fig. 7A) or a significant rescue of etoposide-induced cell death on blocking Kv2 (Supplementary Fig. 7B). It is possible that Kv2 plays a multifaceted and complex role in neurodegeneration, and additional work will be required to reveal if its involvement in amyotrophic lateral sclerosis is via its ion-conducting action or other functions.

While we have identified a novel role for a silent potassium ion channel subunit in driving amyotrophic lateral sclerosis–related neurodegeneration, this does not explain the altered membrane excitability previously identified in SOD1(A4V) MNs. Besides KCNV1, another silent potassium channel subunit, KCNK1, was also downregulated in our study (Fig. 1B). KCNK1 belongs to the two-pore domain potassium channel family that has diverse functions including osteoclastogenesis.^45^ Exactly what drives the altered excitability present in amyotrophic lateral sclerosis MNs and whether ion channel subunits like KCNK1 play a role now need to be addressed, together with discovering how Kv8.1 protects MNs.

## Supplementary Material

fcae202_Supplementary_Data

## Data Availability

Raw and processed RNA-seq data were deposited within the Gene Expression Omnibus repository (https://www.ncbi.nlm.nih.gov/geo/query/acc.cgi? acc=GSE138120).
