# Supplementary material for: Downregulation of the silent potassium channel Kv8.1 increases motor neuron vulnerability in amyotrophic lateral sclerosis
Source: Brain Commun. 2024 Jun 11;6(3):fcae202. doi: 10.1093/braincomms/fcae202 (PMC11191651; doi:10.1093/braincomms/fcae202)
Supplement: fcae202_Supplementary_Data [file fcae202_supplementary_data.zip › Supplementary_Figures.pdf]

Supplementary Figure 1

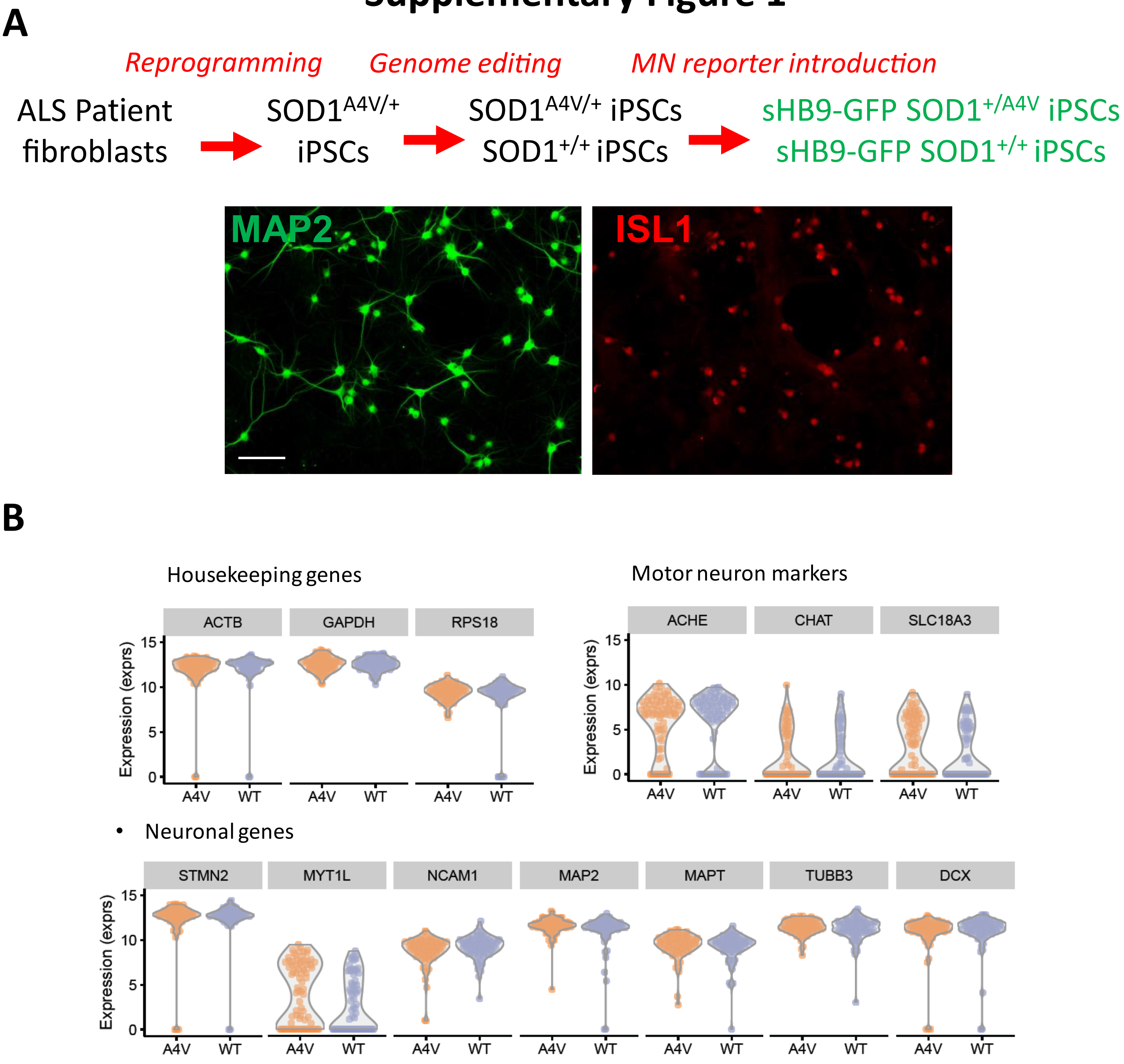

Supplementary Figure 1. Generation of 39b*SOD1*$^{A4V/+}$ and 39b-cor *SOD1*$^{+/+}$ reporter iPS cell lines and their differentiation into motor neurons (MN) for single cell profiling. A, Schematic representation of *SOD1*$^{A4V/+}$ ALS patient iPSC generation, isogenic correction using genome editing, and introduction of the *Hb9* MN reporter. Hb9-GFP positive MNs maintain their identity as indicated by ISL1 expression when co-cultured with mouse primary glial cells for 24 days. (The cells were stained using anti Islet1 and anti MAP2 antibodies. Scale bar, 50 µm)
B, After recording and picking of MNs, cDNA libraries were constructed and sequenced. 181 neurons (N = 43~47 for each 39b and 39b-cor in each two different experimental batch) were analyzed. Violin plot shows that expression of housekeeping genes, motor neuron marker, and neuronal genes is similar between genotypes.

Supplementary Figure 2

Single cell RT-qPCR

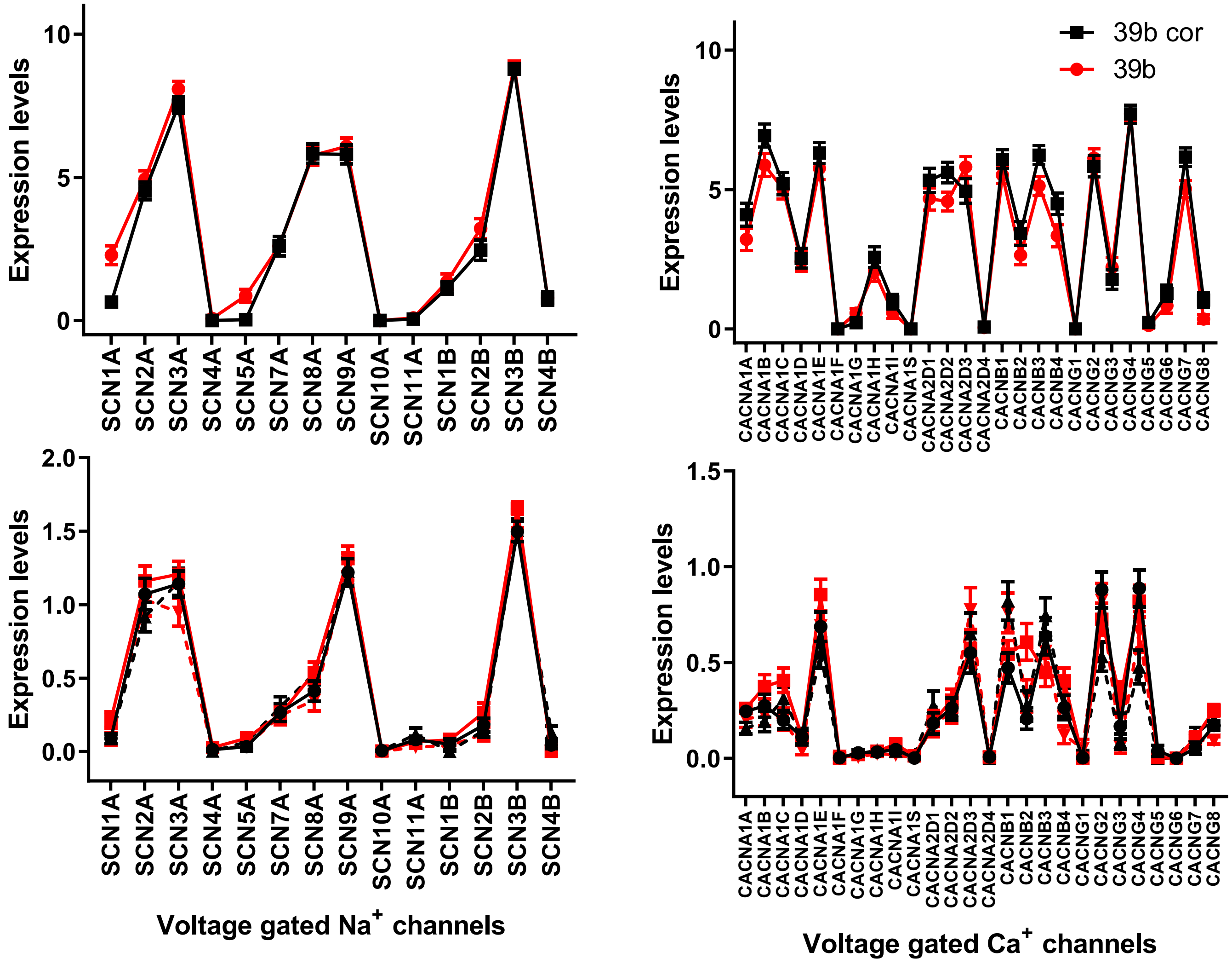

Supplementary Figure 2. Comparison of Na+ and Ca2+ channel expression between 39b *SOD1*$^{A4V/+}$ and 39b-cor *SOD1*$^{+/+}$ MNs determined by single cell RT-PCR. 128 single neurons (N= 64 for 39b and 39b-cor) were analyzed.

Supplementary Figure 3

A

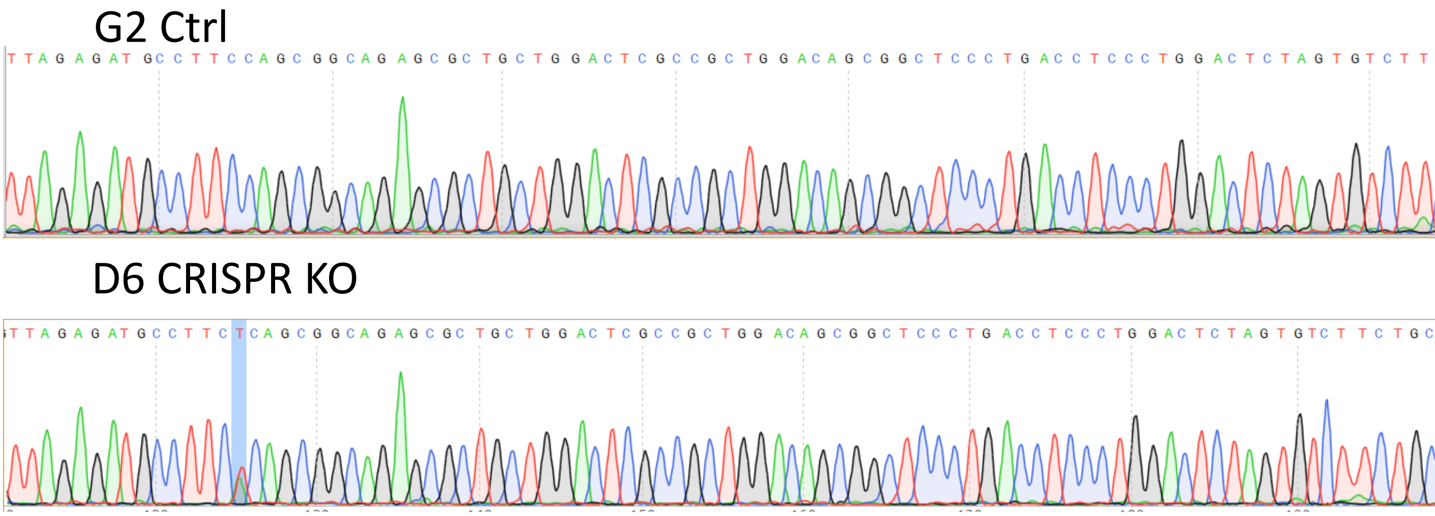

B

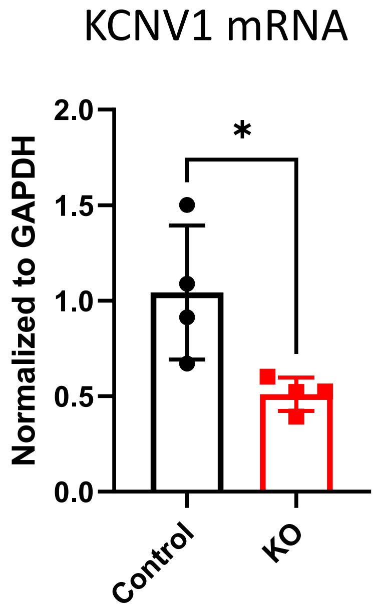

Supplementary Figure 3. Generation of KCNV1 CRISPR KO. A, Sequence of *KCNV1* gene in G2 (control) and D6 (KO) iPSC lines. Single nucleotide insertions were generated in D6 iPSC. B, The expression of *KCNV1* mRNA quantified by qRT-PCR in MNs differentiated from G2 and D6 iPSC (n=4 different culture replicates).

Supplementary Figure 4

A

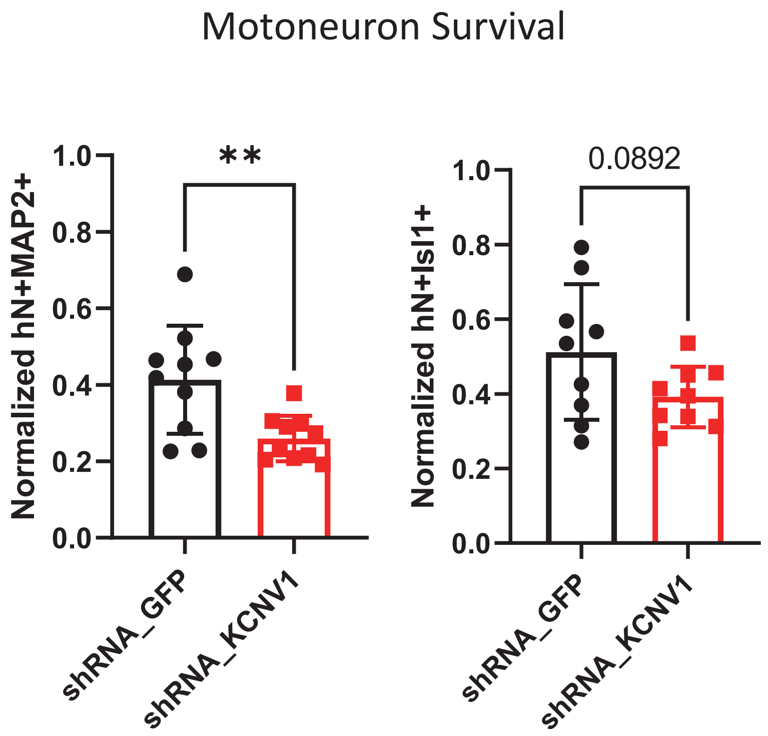

B

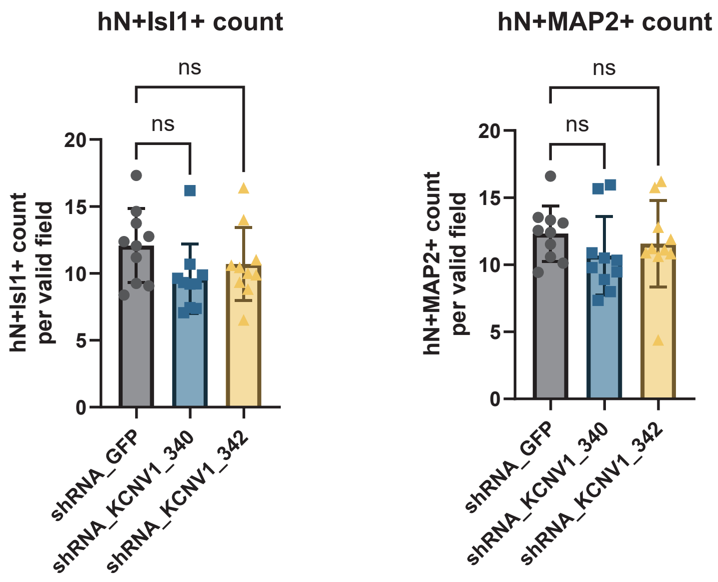

Supplementary Figure 4. A, 39b-cor *SOD1*$^{+/+}$ MNs treated with another *KCNV1* shRNA also show decreased survival after MG-132 treatment (n = 9-10 different coculture replicates from two independent batches of differentiation, with n >= 6 for each batch; average count calculated from more than 10 image fields for each coculture replicate). B, while basal survival is not affected by either shRNA construct. Statistical significances obtained by student's t test (n = 10 different coculture replicates from one batch of differentiation; average count calculated from more than 10 image fields for each coculture replicate) (** P ≤ 0.01).

Supplementary Figure 5

A

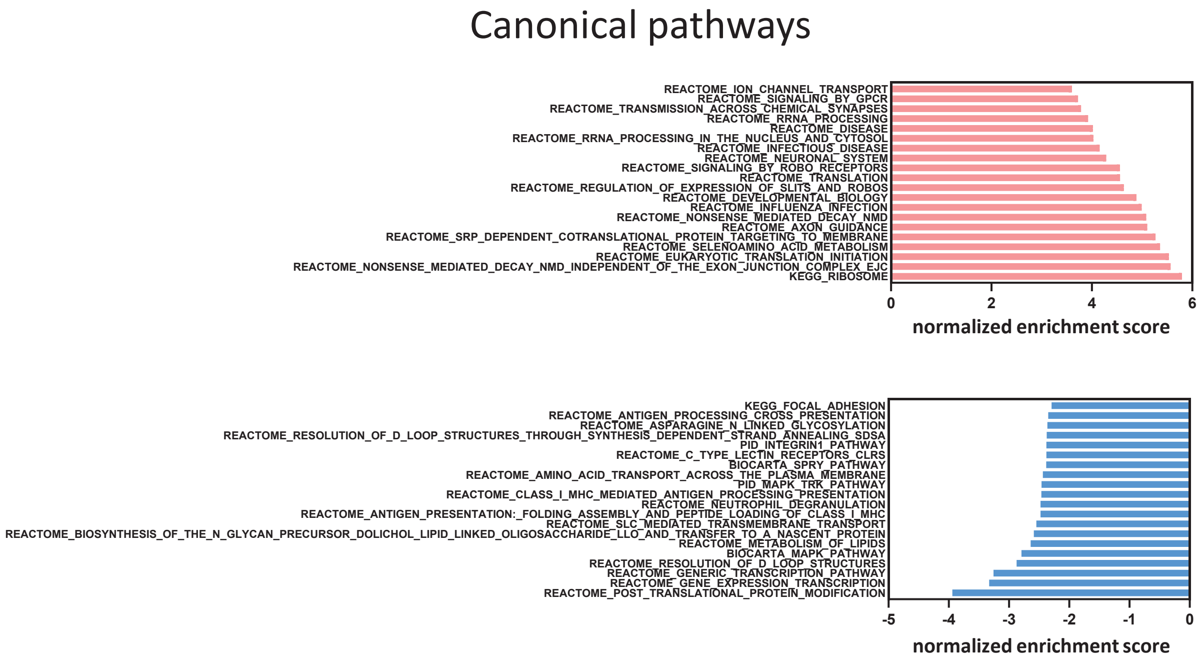

B

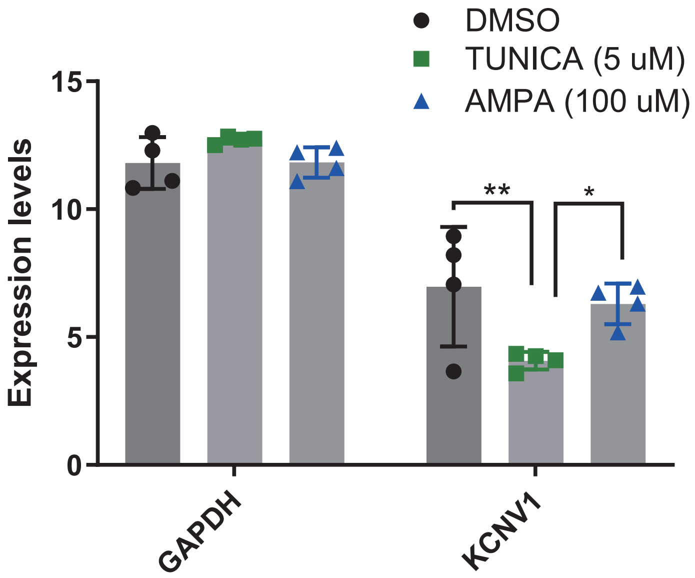

Supplementary Figure 5. A, Enriched pathways in *KCNV1* knock down MNs. Red: up-regulated; Blue: down-regulated. B, Expression of *KCNV1* is reduced by treatment with tunicamycin but not AMPA (n = 4 different coculture replicates).

Supplementary Figure 6

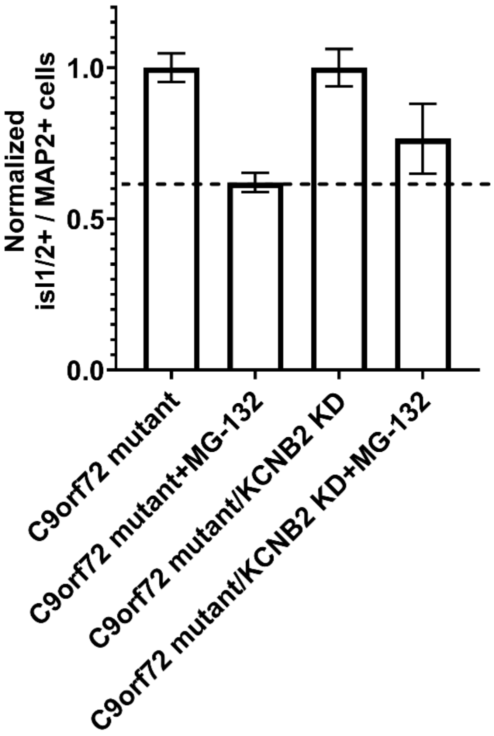

Supplementary Figure 6. Survival of *C9orf72* mutant motor neurons after MG-132 treatment. Motor neurons were quantified by positive staining of ISL1/2 and MAP2 and numbers normalized to DMSO treated control in each group (n=18 different coculture replicates from 3 independent batches of differentiation, with n=6 for each batch; average count calculated from more than 10 image fields for each coculture replicate).

Supplementary Figure 7

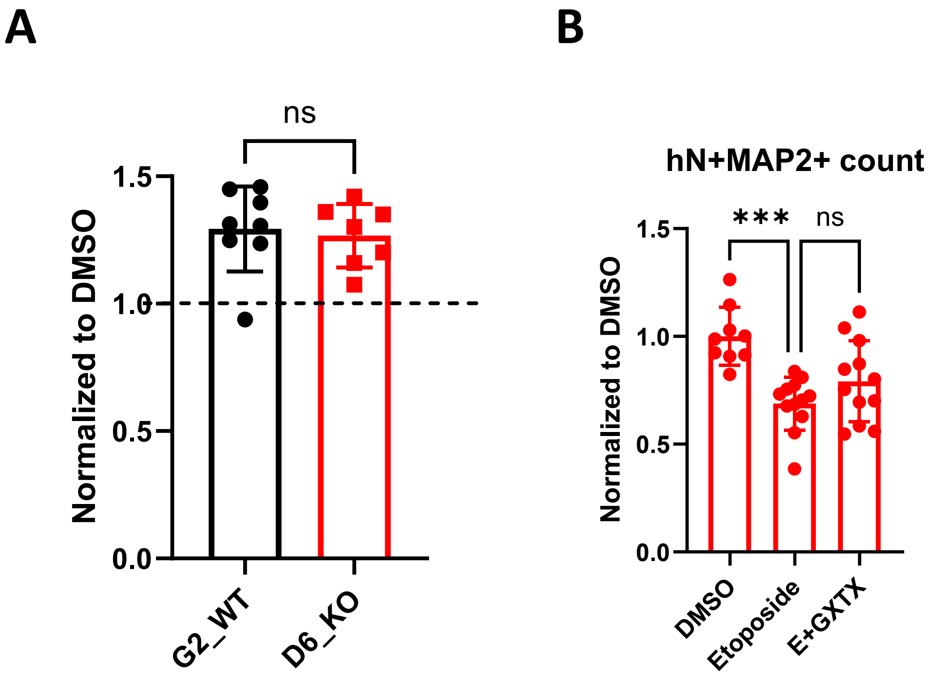

Supplementary Figure 7. A, Normalized action potential firing activity measured by MEA of control and *KCNV1* KO MN cultures 3h after treatment with 0.2 µM GXTX (n= 7-8 different 4-week coculture replicates; average spike rates from 8 electrodes calculated before and after treatment for each coculture). B, Survival of *SOD1*$^{A4V/+}$ ALS MNs after treatment with 10 µM etoposide in the absence or presence of 0.2 µM GXTX (n = 9-12 different coculture replicates from two independent batches of differentiation, with n=3 and 6 for each batch; average count of hN+MAP2+ cells calculated from more than 10 image fields for each coculture replicate).
