# Supplementary material for: Downregulation of the silent potassium channel Kv8.1 increases motor neuron vulnerability in amyotrophic lateral sclerosis
Source: Brain Commun. 2024 Jun 11;6(3):fcae202. doi: 10.1093/braincomms/fcae202 (PMC11191651; doi:10.1093/braincomms/fcae202)
Supplement: fcae202_Supplementary_Data [file fcae202_supplementary_data.zip › Supplementary_table_legends.docx]

**Supplementary Table 1**: Experimental design of single cell patch-seq and patch-qPCR study

**Supplementary Table 2**: List of 63 DEG genes identified from single cell patch-seq study in 39b *SOD1^A4V/+^* and 39b-cor *SOD1^+/+^* MNs

**Supplementary Table 3:** DEG list of bulk-seq collected from 39b-cor *SOD1^+/+^* MNs treated with KCNV1 and control shRNAs
